# Regulation of Microstructure and Absorption Properties of MXene Materials: Theoretical and Experimental

**DOI:** 10.1002/advs.202509994

**Published:** 2025-08-11

**Authors:** Qiang Wang, Xiaolei Su, Yan Jia, Yi Liu, Faisal Shahzad

**Affiliations:** ^1^ School of Materials Science & Engineering Xi'an Polytechnic University Xi'an 710048 P. R. China; ^2^ Research and Innovation Center for Graphene and 2D Materials (RIC2D) Khalifa University P.O. Box 127788 Abu Dhabi UAE

**Keywords:** electromagnetic shielding, electronic band structure, microwave absorption, MXene, transition metals

## Abstract

This study systematically investigates the modulation mechanism of transition metal elements (Ti, Nb, Ta, V) on the microwave absorption performance of MXenes (Ti_3_C_2_T_x_, Ti_2_NbC_2_Tx, Ti_2_TaC_2_T_x_, Ti_2_VC_2_T_x_, Nb_2_CT_x_, V_2_CT_x_). Using multiscale characterization techniques, the microstructure, elemental distribution, and surface chemical states of these materials are comprehensively analyzed. Integrated electromagnetic parameter measurements and theoretical calculations elucidate the physical mechanisms underlying their distinct microwave absorption behaviors. Experimental results reveal that the single‐metal V‐based MXene V_2_CT_x_ exhibits outstanding X‐band (8.2–12.4 GHz) absorption performance, achieving an ultralow RL of −53.8 dB and a broad effective absorption bandwidth of 3.4 GHz. Theoretical calculations indicate that V's multivalent d‐orbitals generate pronounced density of states (DOS) peaks near the Fermi level, significantly enhancing carrier mobility and wave‐carrier interactions. Normalized impedance analysis confirms excellent impedance matching with free space, a critical factor for minimizing microwave reflection and maximizing energy dissipation. In contrast, Ti‐, Nb‐, and Ta‐based MXenes show limited performance, primarily relying on single loss mechanisms that fail to balance impedance and dissipation efficiently. The findings provide theoretical guidance for designing high‐performance broadband microwave absorbers by tailoring atomic‐scale composition to optimize impedance matching and multi‐mechanistic energy dissipation in MXene‐based materials.

## Graphical abstract

This study investigates how transition metals (Ti, Nb, Ta, V) modulate microwave absorption in MXenes. It analyzes their structure and chemistry, finding V‐based MXene excels in X‐band absorption due to unique orbital and microstructure.

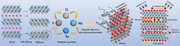

## 1 Introduction

The relentless progress of modern electronic technologies has spurred urgent demands for advanced microwave absorption materials, driven by dual imperatives: mitigating escalating electromagnetic interference (EMI) pollution and enhancing stealth technologies for defense applications.^[^ ^1^, ^2^, ^3^, ^4^ ^]^ In this context, 2D transition metal carbides/nitrides (MXenes) have emerged as transformative candidates, leveraging their unique layered architecture, tunable surface terminations (e.g., ─O, ─F), and metallic conductivity.^[^ ^5^, ^6^, ^7^ ^]^ These attributes endow MXenes with multifaceted microwave loss mechanisms, including conductive dissipation, interfacial polarization, and intra‐layer wave reflections. However, their practical utility is often constrained by suboptimal impedance matching arising from excessive surface wave reflection‐a consequence of their inherently high electrical conductivity.^[^ ^8^ ^]^ For example, pristine Ti_3_C_2_T_x_ exhibits pronounced permittivity mismatch due to its extremely high real permittivity, severely limiting its broadband absorption capacity.^[^ ^9^ ^]^

While early studies primarily focused on Ti‐based MXenes (e.g., Ti_3_C_2_T_x_), recent efforts have expanded to explore MXenes containing other transition metals (TMs). For instance, Nb_2_CT_x_ was reported to exhibit enhanced electrochemical properties due to Nb's d‐band electron configuration, yet its microwave absorption behavior remains underexplored.^[^ ^10^ ^]^ V_2_CT_x_, with unique vanadium redox states, showed promising potential in energy storage, but the correlation between its TM‐derived electronic structure and EMI shielding performance remains unclear.^[^ ^11^ ^]^ Ta‐based MXenes, despite their high melting points and chemical stability, have rarely been investigated for microwave absorption applications.^[^ ^12^ ^]^ Notably, existing research on TM‐doped MXenes has largely centered on compositional modifications (e.g., adding magnetic nanoparticles to Ti_3_C_2_T_x_)^[^ ^13^ ^]^ or structural optimizations (e.g., 3D aerogels),^[^ ^14^ ^]^ with limited focus on how the intrinsic TM species (e.g., Nb, Ta, V) themselves regulate the electronic density of states (DOS), interlayer defects, and carrier mobility‐key parameters governing microwave loss mechanisms. This knowledge gap hinders the rational design of MXenes with optimized absorption performance through atomic‐level TM substitution.

This work systematically investigates six MXene systems (Ti_3_C_2_T_x_, Ti_2_NbC_2_T_x_, Ti_2_TaC_2_T_x_, Ti_2_VC_2_T_x_, Nb_2_CT_x_, V_2_CT_x_) to decipher how TM elements (Ti, Nb, Ta, V) govern microwave absorption mechanisms. Multiscale characterization were employed to correlate crystal structure, surface chemistry, and microstructural evolution with electromagnetic properties. Vector network analyzer measurements of complex permittivity and permeability are integrated with Materials Studio‐based density functional theory (DFT) calculations, revealing how TM substitution modulates band hybridization, DOS, and carrier transport. By establishing the first quantitative correlation between TM species and absorption metrics (Reflection Loss (RL), Effective Absorption Bandwidth (EAB), impedance matching), this study provides a theoretical framework for atomic‐level design of next‐generation MXene‐based absorbers, addressing the critical need to decouple the intrinsic effects of transition metals from composite/structural modifications and enabling precise tuning of microwave absorption through elemental substitution.

## 2 Results and Discussion

### 2.1 Synthesis and Characterization of MXenes

**Figure 1:**
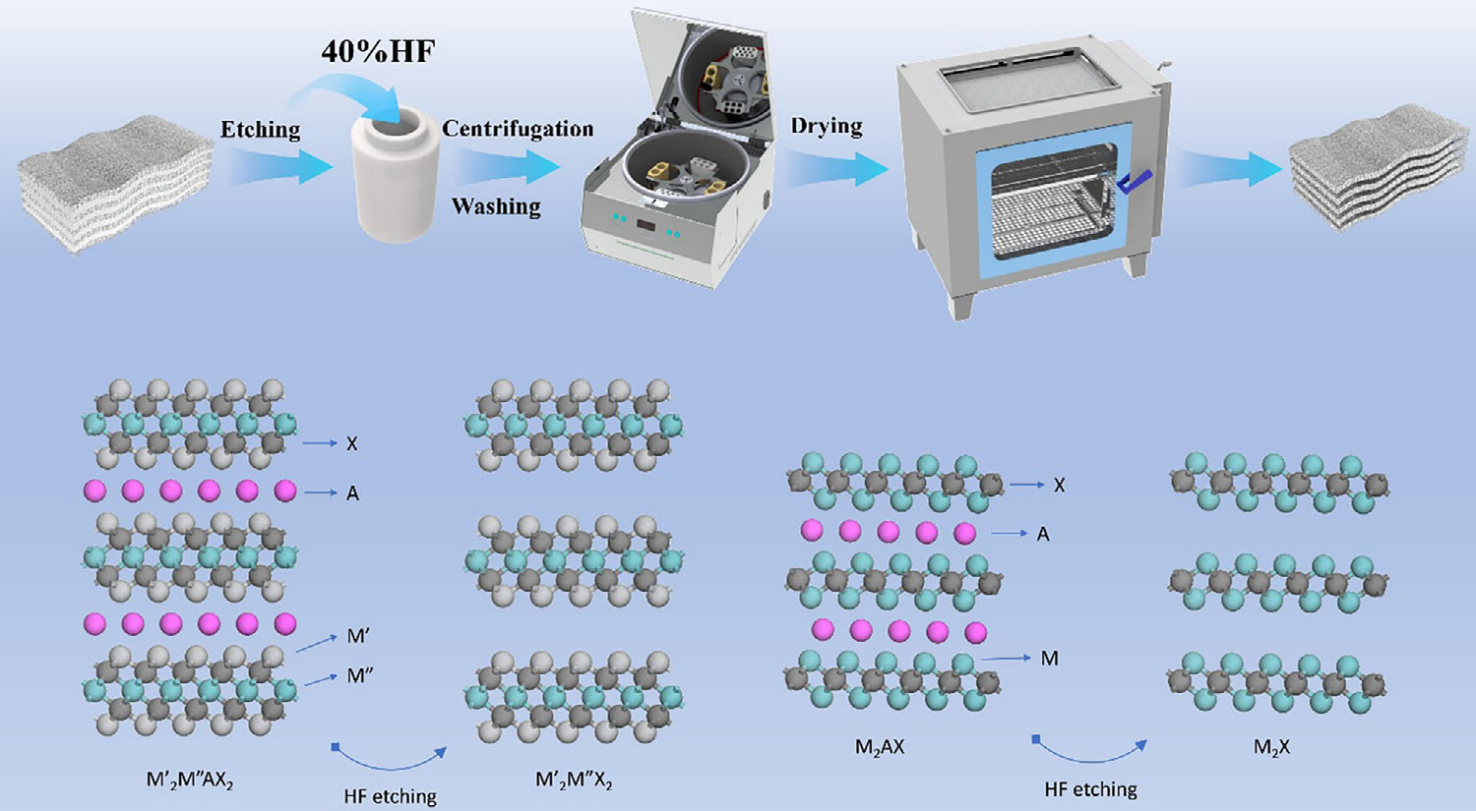
The flow chart of MXene synthesis.

Six transition metal‐doped MXenes (Ti_3_C_2_T_x_, Ti_2_NbC_2_T_x_, Ti_2_TaC_2_T_x_, Ti_2_VC_2_T_x_, Nb_2_CT_x_, V_2_CT_x_) were synthesized via chemical etching of their corresponding aluminum‐containing MAX precursors (Ti_3_AlC_2_, Ti_2_NbAlC_2_, Ti_2_TaAlC_2_, Ti_2_VAlC_2_, Nb_2_AlC, and V_2_AlC, respectively; see the Experimental Section for synthesis details). **Figure** **1** shows the flow chart of MXene synthesis.

**Figure 2:**
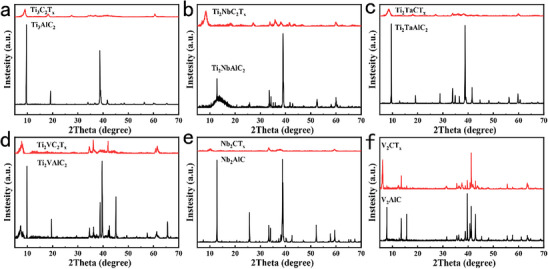
XRD patterns of MXenes before and after etching: a) Ti_3_C_2_T_x_; b) Ti_2_NbC_2_T_x_; c) Ti_2_TaC_2_T_x_; d) Ti_2_VC_2_T_x_; e) Nb_2_CT_x_; f) V_2_CT_x_.

The X‐ray diffraction (XRD) patterns of these MXenes before and after etching are presented in **Figure** **2**a–f. The MAX‐phase precursors (gray curves) exhibit high crystallinity, characterized by a dominant diffraction peak at 2θ = 38.68° along with multiple sharp peaks at higher angles, indicative of their well‐ordered crystalline structures. In contrast, the XRD patterns of the etched MXenes (red curves) reveal substantial structural evolution, as evidenced by the disappearance of MAX‐phase peaks, peak broadening, and reduced intensity. These changes confirm the selective removal of Al layers during etching, which induces layer delamination, increases structural disorder, and diminishes crystallinity, ultimately leading to the characteristic open interlayer morphology of MXenes.^[^ ^12^, ^15^, ^16^ ^]^ Notably, both Ti‐based MXenes (Ti_3_C_2_T_x_, Ti_2_NbC_2_T_x_, Ti_2_TaC_2_T_x_, Ti_2_VC_2_T_x_) and Nb_2_CT_x_/V_2_CT_x_ systems exhibit similar transformation mechanisms. The transition from sharp, high‐intensity MAX‐phase peaks to broadened, low‐intensity MXene peaks visually illustrates the structural evolution associated with Al etching, including layer separation and crystallinity degradation.

**Figure 3:**
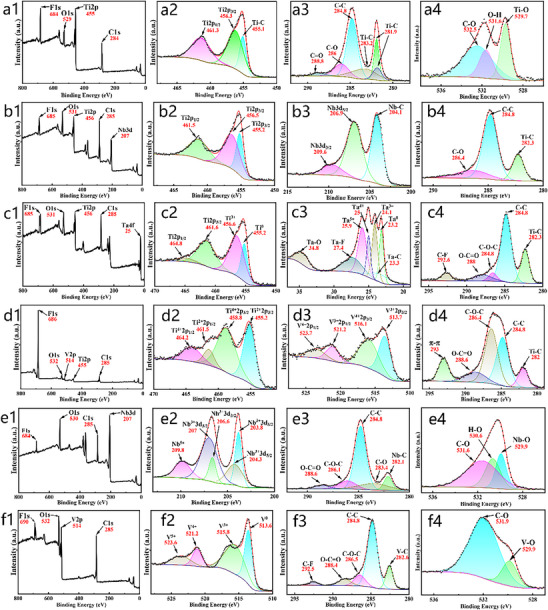
XPS survey and core level spectra: a1–a4) Ti_3_C_2_T_x_; b1–b4) Ti_2_NbC_2_T_x_; c1–c4) Ti_2_TaC_2_T_x_; d1–d4) Ti_2_VC_2_T_x_; e1–e4) Nb_2_CT_x_; f1–f4) V_2_CT_x_.

**Figure** **3**a–f presents a detailed X‐ray photoelectron spectroscopy (XPS) analysis of six MXenes, unraveling the atomic‐scale interplay between metal doping, surface functional groups, and electronic structure. The full XPS spectrum (3a1) exhibits distinct peaks for Ti2p (455 eV), C1s (285 eV), O1s (529 eV), and F1s (684 eV), confirming the presence of Ti, C, O, and surface‐adsorbed F groups. The Ti2p_3/2_ peak is located at 456.3 eV, and the Ti2p_1/2_ peak appears at 461.3 eV (3a2). These are consistent with the characteristic peak positions of Ti^3^⁺, indicating the presence of Ti^3+^. The Ti─C bond (455.1 eV) suggests that Ti forms a covalent bond with C. This is a typical bonding form in MXene materials, illustrating that Ti is in an environment combined with C and participates in the formation of the Ti─C skeleton structure. The C1s spectrum (3a3) shows coexisting C─C (284.8 eV) and C─O (286.5 eV) bonds, reflecting the oxidation state diversity on the carbon layers, while the F1s signal (3a1) highlights the inherent surface fluorination in pristine Ti‐based MXenes. The Nb3d doublet (206.9, 209.6 eV, 3b3) corresponds to Nb^3^⁺, confirming lattice incorporation via strong Nb─C covalent bonds. The Ti2p spectrum (3b2) displays mixed Ti^2+^/Ti^3+^ valence states, attributed to Nb's lower electronegativity modulating the Ti electron cloud density. A reduced intensity of C─O bonds in the C1s spectrum (3b4) indicates that Nb doping suppresses surface oxidation, potentially enhancing chemical stability by minimizing hydrophilic oxygen‐containing groups. The Ta4f region (3c3) features a prominent Ta^5+^ signal with a characteristic Ta─C bonding peak, evidencing Ta participation in lattice coordination. A C─F bond peak at 288.0 eV in the C1s spectrum (3c4) arises from Ta‐induced surface fluorination, which modulates surface polarity. V2p spectra (3d3) reveal coexisting V^3+^ (513.7, 521.2 eV) and V^4+^ (516.1, 523.7 eV), demonstrating V's redox activity and ability to act as an electron acceptor, as corroborated by the Ti^2+^ peak (455.2 eV, 3d2) forming a Ti^2+^─V^3+^ charge‐compensation pair. The intense F1s peak (686 eV, 3d1) stems from multiple factors: 1) synthesis‐induced fluorination using HF‐based etchants, which preferentially adsorb at V sites due to their higher charge density (V^4+^); 2) defect‐rich surfaces providing additional adsorption sites for F^−^; and 3) XPS sensitivity to surface‐enriched species. Despite the strong F signal, the dominant Ti─C and V─C covalent bonds in the main structure remain intact, with surface C─F and carbonyl groups (O─C═O, 288.6.0 eV, 3d4) enhancing polar interfaces for improved dielectric loss. Nb3d peaks (203.8, 204.3, 206.6, and 207 eV, 3e2) confirm Nb^3^⁺ in the lattice, while the C1s spectrum (3e3) is dominated by C─C bonds (284.8 eV) with reduced C─O content, indicating a low‐oxidation surface state in Ti‐free Nb_2_C. The C1s spectrum (3f3) exhibits high‐intensity C─F bonds (292.5 eV) due to V‐induced deep fluorination, driven by V's strong reducibility that suppresses surface oxidation. This high‐fluorination, low‐oxidation surface state enhances dipole polarization through strong C─F bonds, critical for optimizing microwave absorption.

Electronegativity disparities among doped metals (Nb, Ta, V) dictate distinct valence state modulations: Nb/Ta lower Ti oxidation via electron cloud adjustment, while V forms redox pairs (V^3+^/V^4+^‐Ti^2+^) to enhance electronic structure complexity. Surface functional group evolution is equally critical: Ti‐based MXenes exhibit mixed O/F terminations, Nb/Ta doping increases F‐containing groups to tune surface polarity, and V‐based systems achieve high fluorination due to V's affinity for F^−^. Bonding characteristics‐covalent Ti─C, robust Nb/Ta─C, and spin‐polarized V─C interfaces‐collectively regulate electron transport and polarization mechanisms. These XPS insights establish a structural basis for MXene absorption performance, highlighting how doping‐induced valence mixing, functional group reconstruction, and interfacial polarity synergistically enhance dielectric loss and polarization relaxation, pivotal for advanced electromagnetic applications.

**Figure 4:**
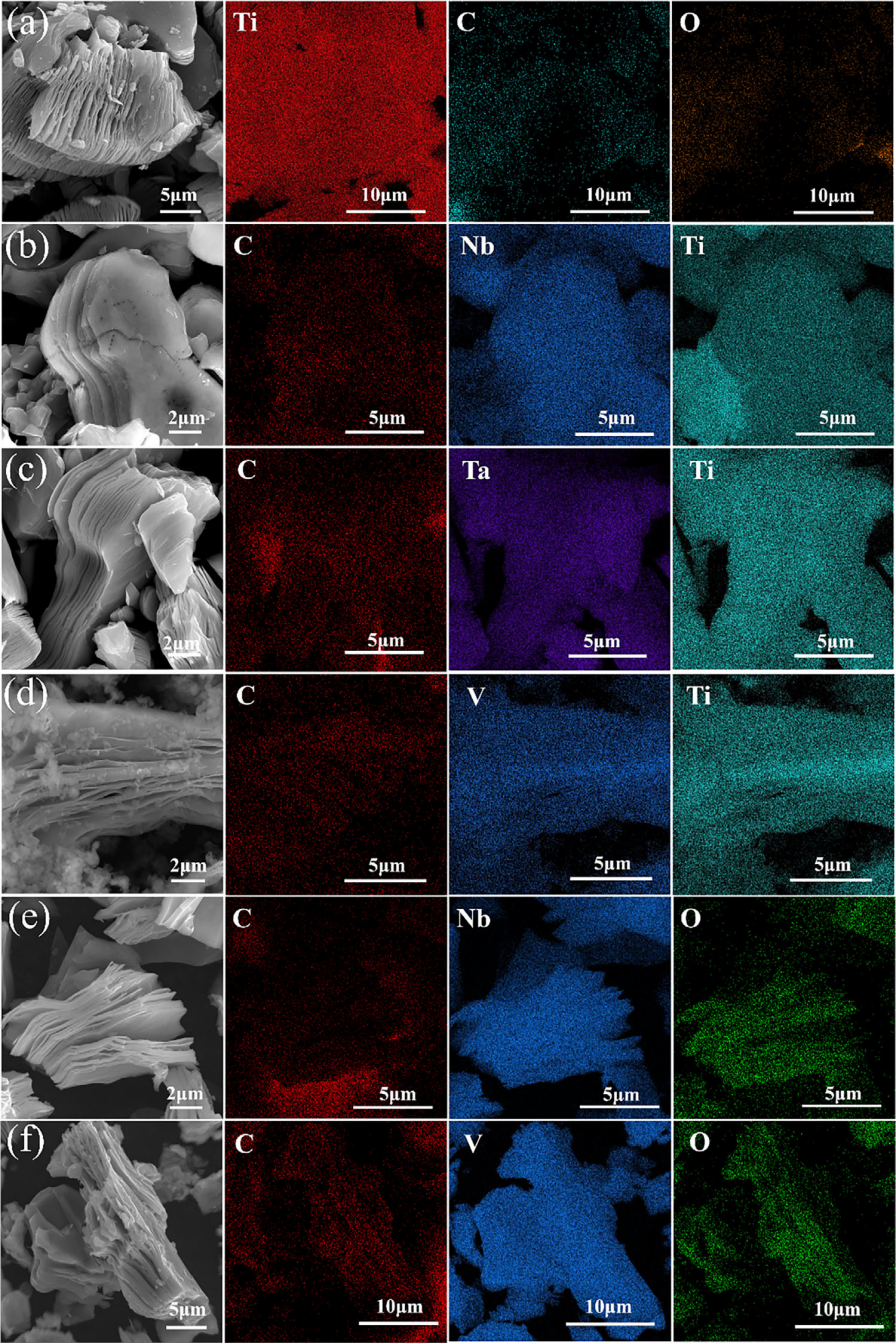
SEM images and EDS elemental mappings of MXenes: a) Ti_3_C_2_T_x_; b) Ti_2_NbC_2_T_x_; c) Ti_2_TaC_2_T_x_; d) Ti_2_VC_2_T_x_; e) Nb_2_CT_x_; f) V_2_CT_x_.

**Figure** **4** displays SEM images and EDS elemental mappings of six MXenes, offering critical insights into their structural characteristics and their potential correlations with microwave absorbing properties. For Ti_3_C_2_T_x_ (4a), the SEM image reveals a typical layered morphology, providing a large specific surface area that facilitates interfacial polarization – a key mechanism for microwave absorption. EDS mappings show uniform Ti distribution, with C forming the structural backbone and O heterogeneously distributed, likely from surface – bound oxygen – containing functional groups that introduce additional polar centers, enhancing dielectric polarization for electromagnetic energy dissipation. Ti_2_NbC_2_T_x_ (4b) exhibits a well – defined layered structure with smooth – surfaced flakes in the SEM image. The regular layering aids ordered electron transport and interface formation. EDS confirms uniform C distribution, with Nb dispersion adjusting Ti's electron – cloud density to optimize impedance matching – essential for efficient microwave absorption. The homogeneous Ti distribution ensures a consistent electronic environment. In Ti_2_TaC_2_T_x_ (4c), the SEM shows a layered morphology with slightly wrinkled flakes, increasing surface and interface complexity for more electromagnetic wave scattering and absorption pathways. EDS indicates uniform C distribution, while Ta doping alters interlayer electronic coupling via strong covalent bonds, enhancing dielectric loss and converting electromagnetic energy into heat. The homogeneous Ti presence ensures stable electromagnetic performance. Ti_2_VC_2_T_x_ (4d) displays an exfoliated, thread – like layered structure in the SEM, exposing more edges and defects as active polarization sites. EDS shows uniform C distribution, and V's redox activity forms charge – compensation pairs (e.g., Ti^2+^‐V^3+^), enhancing interfacial and dipolar polarization for efficient energy dissipation through multiple relaxation processes. Nb_2_CT_x_ (4e) presents a stacked, flake – like layered structure in the SEM, where regular stacking influences interlayer spacing and electronic coupling, affecting dielectric properties. EDS shows uniform C distribution, with Nb's electronic configuration enabling specific electron – transport pathways for tuned dielectric loss. V_2_CT_x_ (4f) shows a layered morphology with mixed flake sizes in the SEM, creating a hierarchical structure for multi – scale electromagnetic wave scattering and enhanced absorption efficiency. The EDS section in the figure demonstrates the distribution characteristics of C, V, and O elements in V_2_CT_x_. C is uniformly distributed, permeating the entire material as a fundamental component of the V_2_CT_x_ lattice; V is evenly dispersed, confirming the successful and uniform incorporation of vanadium into the structure, ensuring the consistency of the material composition; O is unevenly distributed, presumably originating from surface oxygen – containing functional groups (such as ─F ─O) or local oxidation, reflecting the complexity of the material's surface chemical environment. EDS technology determines the types and distributions of elements by detecting characteristic X–rays. In this figure, it provides critical information for the analysis of compositional uniformity and surface chemical states of MXenes, aiding in the in – depth understanding of the correlation between material structure and properties.

In summary, the structural features (layered morphology, flake size, defects) and elemental distributions (doping elements, oxygen – containing groups) of these MXenes significantly influence their electromagnetic – wave – absorbing properties. The layered structure provides a foundation for interfacial polarization, doping elements adjust electronic structures to optimize dielectric loss and impedance matching, and surface – bound groups introduce additional polarization mechanisms. These factors collectively determine how each MXene interacts with electromagnetic waves, guiding the design of high – performance wave – absorbing materials.

**Figure 5:**
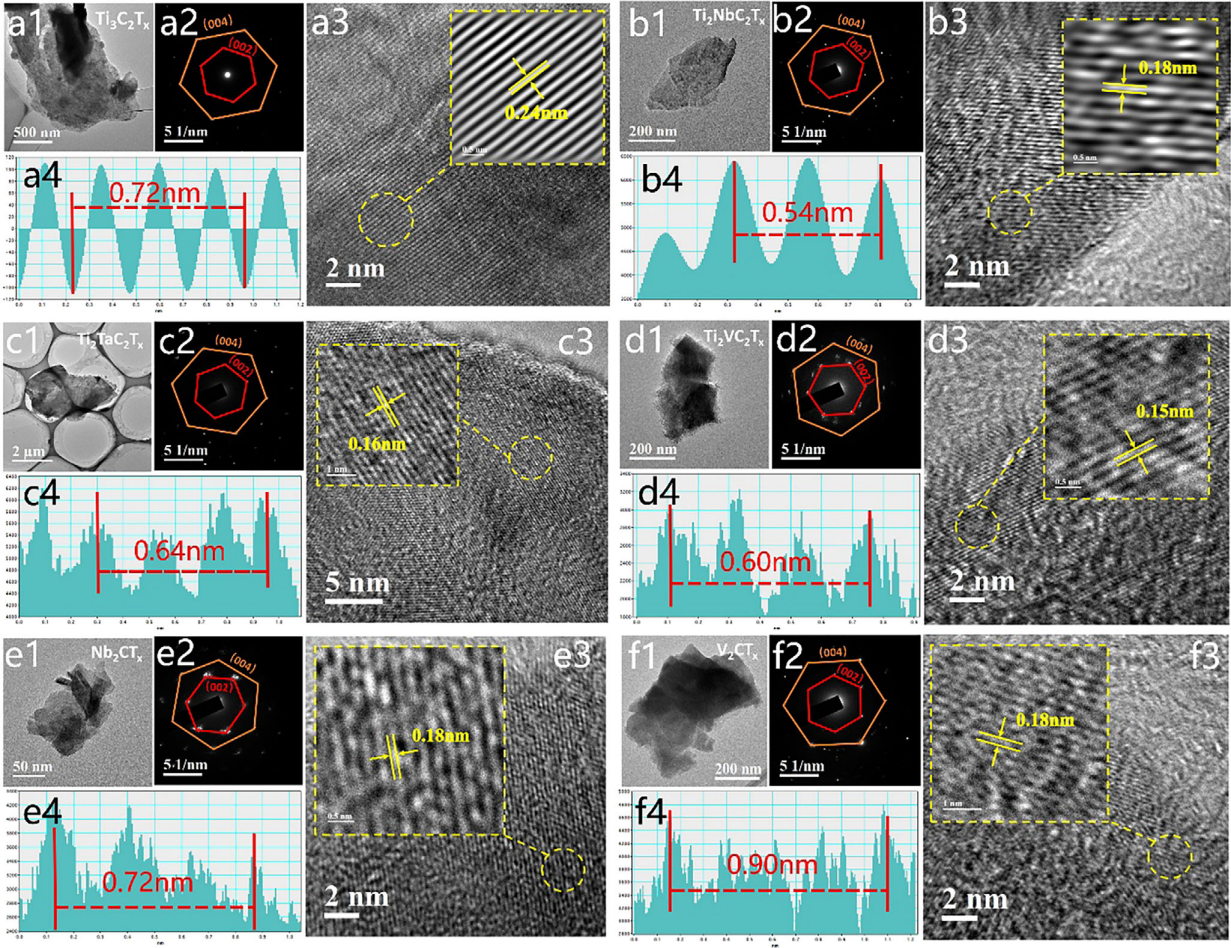
TEM. a1) Ti_3_C_2_T_x_, b1) Ti_2_NbC_2_T_x_, c1) Ti_2_TaC_2_T_x_, d1) Ti_2_VC_2_T_x_, e1) Nb_2_CT_x_, f1) V_2_CT_x_; Selected‐area electron diffraction (SAED) patterns: a2) Ti_3_C_2_T_x_, b2) Ti_2_NbC_2_T_x_, c2) Ti_2_TaC_2_T_x_, d2) Ti_2_VC_2_T_x_, e2) Nb_2_CT_x_, f2) V_2_CT_x_; High‐resolution TEM (HRTEM): a3) Ti_3_C_2_T_x_, b3) Ti_2_NbC_2_T_x_, c3) Ti_2_TaC_2_T_x_, d3) Ti_2_VC_2_T_x_, e3) Nb_2_CT_x_; f3) V_2_CT_x_; Line – profile plot: a4) Ti_3_C_2_T_x_, b4) Ti_2_NbC_2_T_x_, c4) Ti_2_TaC_2_T_x_, d4) Ti_2_VC_2_T_x_, e4) Nb_2_CT_x_.

**Figure** **5** demonstrates a multidimensional structural characterization of six MXenes using transmission electron microscopy (TEM), high – resolution TEM (HRTEM), and selected – area electron diffraction (SAED), providing critical insights into the structural evolution of transition – metal – doped MXenes and their implications for microwave absorbing properties. For Ti_3_C_2_T_x_, the TEM image (Figure 5a1) confirms its typical 2D layered morphology. The HRTEM (Figure 5a3) reveals well – defined lattice fringes corresponding to the (002) plane of a hexagonal crystal system, and the SAED pattern (Figure 5a2) exhibits distinct hexagonal symmetry, indicating high crystallinity. The lattice spacing in this regular structure influences electron – cloud distribution and polarization, affecting the dielectric constant – a key parameter for wave – absorbing performance. A stable lattice structure provides a foundation for consistent electromagnetic response, optimizing impedance matching to some extent. In Ti_2_NbC_2_T_x_, the TEM image (Figure 5b1) exhibits a layered structure, while Nb doping induces lattice compression due to atomic‐radius mismatch, as evident from the reduced lattice‐fringe spacing in HRTEM (Figure 5b3) – decreasing from 0.24 nm in Ti_3_C_2_T_x_ to 0.18 nm in Ti_2_NbC_2_T_x_, which directly reflects the structural modification caused by Nb incorporation. The SAED pattern (Figure 5b2) maintains hexagonal symmetry with slight spot shifts. This lattice compression alters the electronic structure, modifying polarization behavior. Such changes adjust the material's impedance matching and attenuation capacity, which are essential for wave absorption. The reduced spacing can enhance certain polarization mechanisms, potentially improving energy dissipation of electromagnetic waves. Figure 5c1 depicts the morphology of Ti_2_TaC_2_T_x_. Due to the comparable atomic size of Ta and Ti, lattice integrity is well‐preserved, as demonstrated by continuous HRTEM fringes (Figure 5c3) and clear SAED spots (Figure 5c2) with only minor lattice‐parameter adjustments, indicating that Ta incorporation induces subtle structural perturbations without compromising crystallinity. Although the lattice distortion is minimal, subtle changes in lattice spacing still affect electron – phonon interactions and polarization relaxation. These effects influence the wave – absorbing frequency band and efficiency, as even small structural modifications can alter the material's electromagnetic response. Figure 5d1 exhibits the TEM image of Ti_2_VC_2_T_x_’s MXene. The smaller atomic radius of V induces localized lattice distortions, evident from the twisted HRTEM fringes (Figure 5d3) and diffuse SAED scattering (Figure 5d2). These distortions introduce abundant defects and interface polarizations. The additional polarization mechanisms enhance electromagnetic wave energy dissipation. By increasing attenuation through multiple polarization relaxation processes, the material's wave – absorbing performance is improved, expanding its potential application in absorbing a broader range of electromagnetic waves. Nb_2_CT_x_’s TEM image (Figure 5e1) reveals thin, curled edges. The HRTEM (Figure 5e3) shows tightly spaced fringes, and the SAED pattern (Figure 5e2) is hexagonal, indicating a hexagonal structure with high crystallinity and minimal lattice distortion. The tight lattice spacing modulates the dielectric response, affecting the material's interaction with electromagnetic waves. While a well – defined lattice structure can optimize impedance matching, the limited defect – related polarization may restrict the broadening of absorption bands. Lastly, Figure 5f1 shows the TEM image of V_2_CT_x_’. The HRTEM (Figure 5f3) fringes are distorted, and the SAED pattern (Figure 5f2) is highly diffuse, suggesting severe structural disorder. The pronounced lattice irregularities and stacking defects create abundant interfaces and defects, leading to strong interface polarization and defect – related relaxation. This significantly enhances the material's capacity to dissipate electromagnetic energy, potentially broadening the absorption bandwidth and improving overall wave – absorbing efficiency.

In conclusion, the structural characterizations via TEM, HRTEM, and SAED for these six MXenes highlight the profound influence of transition – metal doping on their structural properties. Lattice spacing and fringe changes alter electronic structures, polarization mechanisms, and defect states, which are crucial for tuning electromagnetic parameters (such as dielectric constant and loss tangent). These adjustments directly impact wave – absorbing performance, including impedance matching, attenuation capacity, and absorption bandwidth.^[^ ^17^, ^18^, ^19^ ^]^

### 2.2 Electronic Structure of MXenes

**Figure 6:**
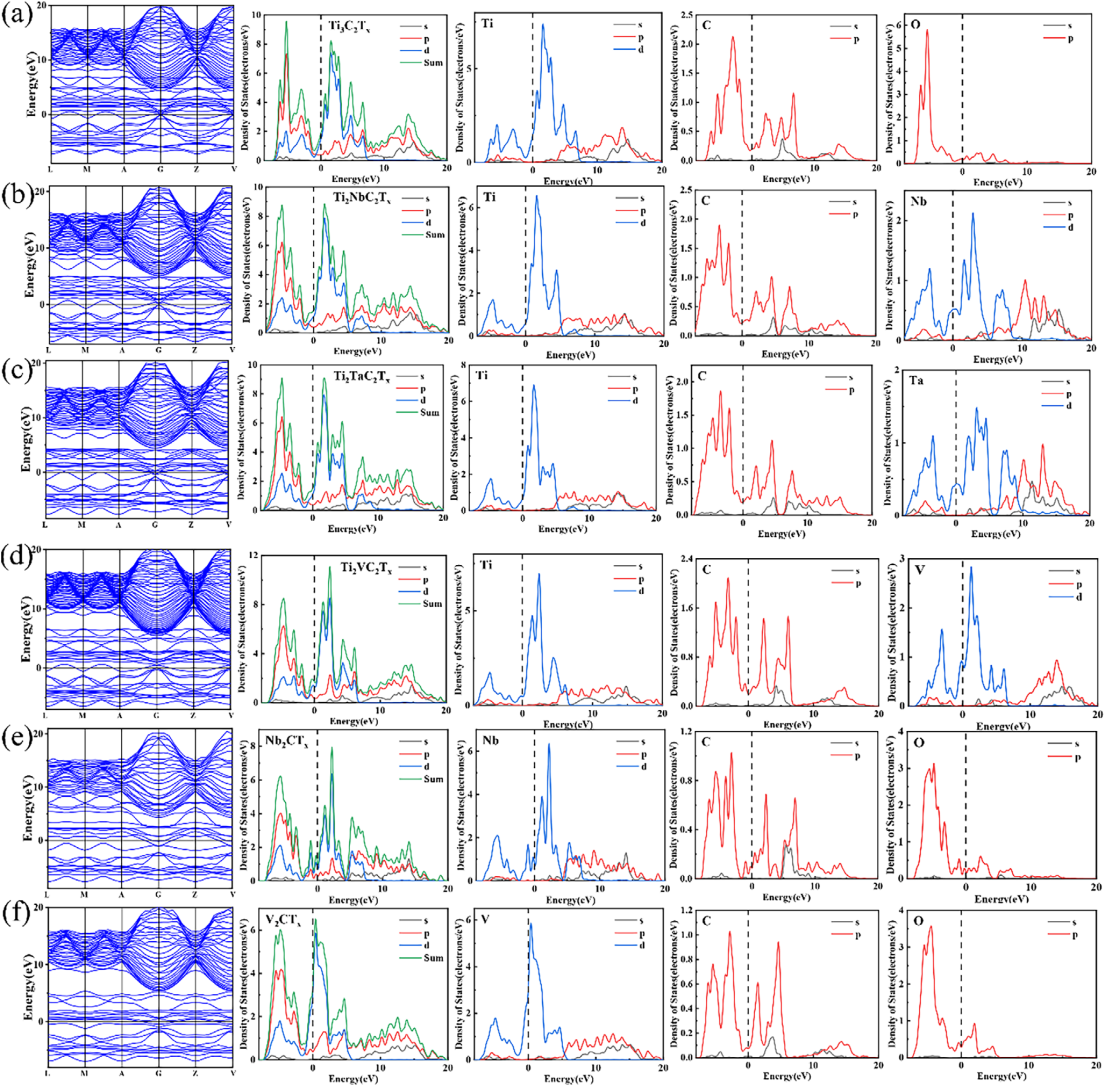
Band structures, total density of states (DOS), and partial density of states (PDOS) plots: a) Ti_3_C_2_T_x_; b) Ti_2_NbC_2_T_x_; c) Ti_2_TaC_2_T_x_; d) Ti_2_VC_2_T_x_; e) Nb_2_CT_x_; f) V_2_CT_x_.

**Figure** **6** comprehensively analyzes the electronic band structures and density of states (DOS) of six MXenes, elucidating how orbital contributions and atomic – size effects dictate their microwave – absorption behavior. For Ti_3_C_2_T_x_ (Figure 6a), its metallic nature, confirmed by bands intersecting the Fermi level, enables easy carrier transport. The DOS at the Fermi level, dominated by Ti d – orbitals, drives conductive loss. Carbon p – orbitals reinforce the 2D lattice via covalent bonding, optimizing electron delocalization, while oxygen p – orbitals from surface terminations create interfacial polarization centers for dielectric loss. In Ti_2_NbC_2_T_x_ (Figure 6b), Nb d – orbitals hybridize with Ti d – states, redistributing DOS near the Fermi level. Although Ti remains the main carrier pathway, Nb‐induced hybridization subtly adjusts conductive – loss intensity. Nb's atomic radius (1.46 Å) promotes localized charge accumulation at interfaces, activating polarization loss, yet limited overlap between conductive and dielectric mechanisms restricts overall absorption enhancement.^[^ ^20^, ^21^, ^22^, ^23^ ^]^ For Ti_2_TaC_2_T_x_ (Figure 6c), the d – orbitals of Ta hybridize with the d – states of Ti, leading to the dispersion of the density of states (DOS) peaks. Given that Ta has an atomic radius of 1.44 Å, which is smaller than that of Ti (1.47 Å), it causes minor lattice compression. This lattice compression reduces carrier mobility and weakens conductive loss. While orbital overlap creates new polarization centers, single – element doping limits synergistic loss improvement. Ti_2_VC_2_T_x_ (Figure 6d) benefits from vanadium's smaller atomic radius (1.32 Å), which induces lattice contraction that significantly enhances the orbital overlap between V and Ti atoms. This structural adjustment promotes strong multivalent d‐orbital hybridization, reshaping the electronic band structure to enable efficient carrier transport and broadened electron delocalization across the 2D lattice. The enhanced band hybridization not only facilitates high carrier mobility but also strengthens the material's capacity for electromagnetic wave coupling, allowing incident microwaves to interact more effectively with the conductive electron gas. Concurrently, the lattice contraction and atomic‐scale strain introduced by vanadium drive the formation of a defect‐rich microstructure, where vacancies, edge terminations, and uneven atomic arrangements act as interfacial polarization centers. Under microwave excitation, these centers generate dynamic dielectric responses, such as dipolar rotation and interfacial polarization, which contribute to substantial dielectric loss. The synergistic interplay between the hybridization‐induced carrier‐wave interaction and defect‐mediated dielectric polarization creates a dual‐mechanism framework in Ti_2_VC_2_T_x_, enabling tunable and high‐efficiency microwave absorption across a broad frequency range.^[^ ^24^, ^25^, ^26^, ^27^ ^]^ Nb_2_CT_x_ (Figure 6e) shows a DOS overwhelmingly dominated by Nb d – orbitals, yielding high carrier density. However, the lack of multiple transition – metal species limits polarization – loss contributions due to fewer defects, resulting in weaker absorption modulation. V_2_CT_x_ (Figure 6f) exhibits the highest metallicity among the studied MXenes, evident from its sharp V d‐orbital density of states (DOS) peaks at the Fermi level. This electronic configuration facilitates efficient electron delocalization across the 2D lattice, enabling strong coupling with incident microwaves through collective carrier oscillations. Vanadium's multivalence (e.g., V^3+^/V^4+^) induces abundant structural defects‐including atomic vacancies, interlayer misalignments, and uneven terminations‐while surface oxygen p‐orbitals introduce polarizable functional groups. These defects and surface terminations act as critical interfacial polarization centers, generating dynamic dielectric responses under microwave excitation. Under alternating electromagnetic fields, the densely packed V d‐orbitals and defect‐rich microstructure promote two primary loss mechanisms: wave‐carrier interactions (mediated by delocalized electrons in the metallic band structure) and dielectric polarization (driven by rotational/relaxational motions of polar groups and space‐charge accumulation at defect interfaces). The former converts microwave energy into lattice vibrations via electron‐phonon scattering, while the latter dissipates energy through dipolar rotations and interfacial charge redistribution. Overall, the analysis highlights how atomic‐scale features (e.g., V's multivalence and orbital hybridization) and microstructural defects systematically regulate polarization loss mechanisms in MXenes, offering critical design principles for next‐generation microwave‐absorbing materials.

### 2.3 Microwave Wave Absorption Performance of MXenes

**Figure 7:**
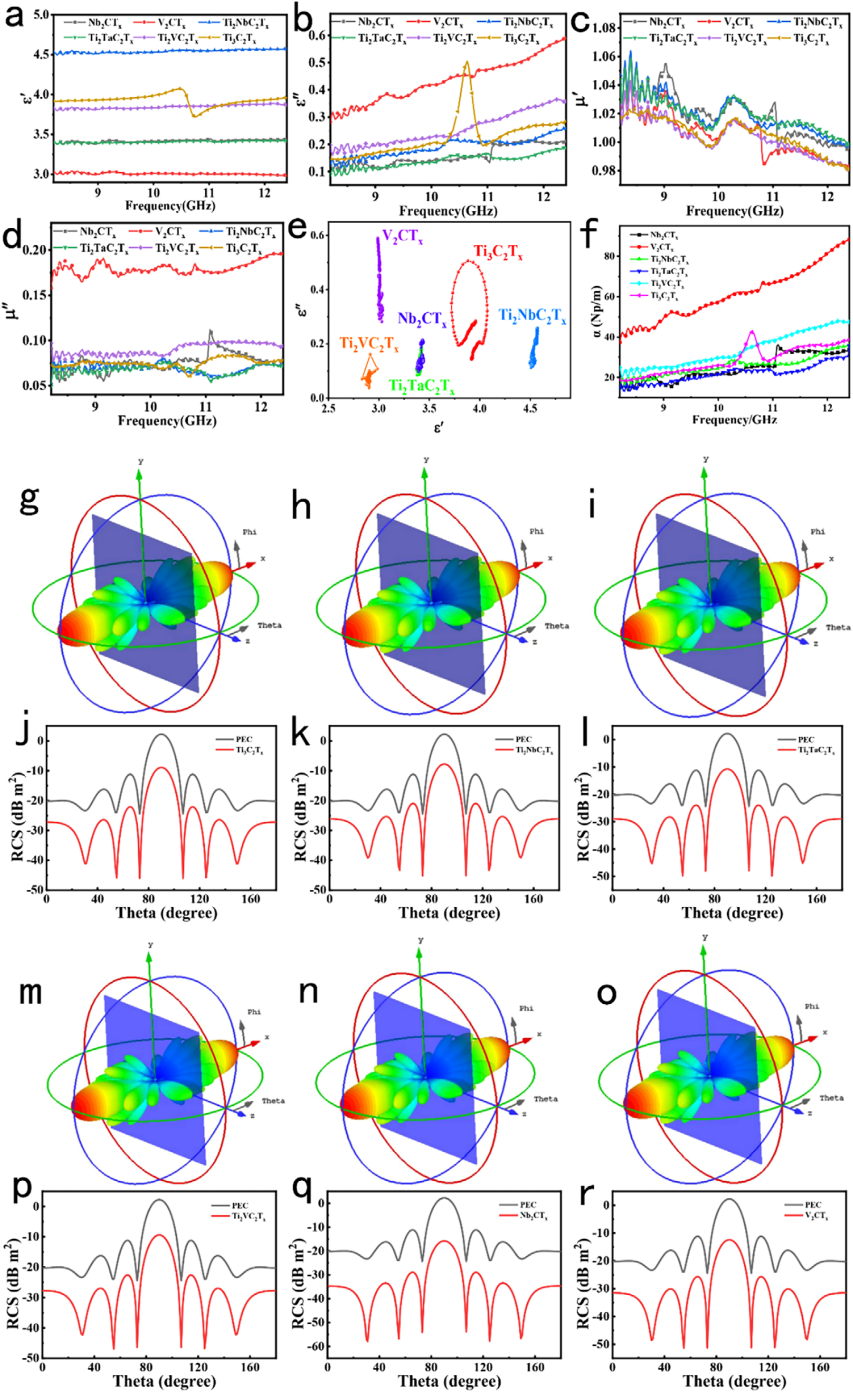
a) Real permittivity (ε′); b) Imaginary permittivity (ε′′); c) Real permeability (µ′); d) Imaginary permeability (µ′′); e) Cole‐Cole plots; f) Attenuation coefficient (α). Far‐field radiation patterns: g) Ti_3_C_2_T_x_; h) Ti_2_NbC_2_T_x_; i) Ti_2_TaC_2_T_x_; m) Ti_2_VC_2_T_x_; n) Nb_2_CT_x_; o) V_2_CT_x_. Radar cross‐section (RCS) frequency responses: j) Ti_3_C_2_T_x_; k) Ti_2_NbC_2_T_x_; l) Ti_2_TaC_2_T_x_; p) Ti_2_VC_2_T_x_; q) Nb_2_CT_x_; r) V_2_CT_x_.

The electromagnetic parameters of MXene‐paraffin composites (15 wt.% MXene) were measured using a vector network analyzer. **Figure** **7**a–f summarize the key results. In Figure 7a, the real permittivity (ε′) of the six MXenes across the X‐band (8.2‐12.4 GHz) reveals distinct dielectric responses. Ti_2_NbC_2_T_x_ exhibits the highest ε′ (4.5), attributed to Nb‐induced optimization of the electronic structure and enhanced interfacial polarization, while V_2_CT_x_ shows the lowest ε′ (3.0), reflecting suppressed dielectric storage arising from its inherent single‐metal V framework and delocalized d‐orbital electronic structure. Ti_3_C_2_T_x_ displays intermediate ε′ (3.8–4.0), characteristic of its intrinsic dielectric behavior. The ε′ values of Ti_2_TaC_2_T_x_, Ti_2_VC_2_T_x_, and Nb_2_CT_x_ fall between these extremes, reflecting transition metal‐dependent modulation of interfacial/dipolar polarization and electronic structure. Figure 7b highlights the dielectric loss (ε′′). V_2_CT_x_ achieves the highest ε′′ (0.6 at high frequencies) due to synergistic multivalent polarization, defect‐induced polarization, and interfacial polarization from surface functional groups. Ti_3_C_2_T_x_ exhibits a prominent ε′′ peak linked to structural relaxation, while other MXenes show moderate losses governed by doping‐specific mechanisms. The real permeability (µ′) in Figure 7c fluctuates minimally (0.98–1.08) for all MXenes, confirming negligible magnetic energy storage, consistent with the non‐magnetic nature of MXenes.^[^ ^28^, ^29^, ^30^, ^31^ ^]^ The magnetic loss (µ′′, Figure 7d) remains below 0.2, with slight increases in V_2_CT_x_ at high frequencies, further supporting dielectric‐dominated absorption mechanisms. The Cole‐Cole curves (Figure 7e) of the six types of MXene materials show significant morphological differences, which directly reflect the essential distinctions in their dielectric loss mechanisms and microwave absorption properties. The curve of Ti_3_C_2_T_x_ is approximately semicircular but with distorted edges, indicating that a single dielectric relaxation process dominates inside it. Due to the constraint of the main relaxation mechanism, the effective microwave absorption bandwidth is relatively narrow. The Ti_2_NbC_2_T_x_ exhibits the characteristic of multi‐segment scattered arcs, reflecting the existence of multiple dielectric relaxation times in the material. Through the synergistic effect of multiple mechanisms such as interface polarization, dipole rotation polarization, and electronic conduction, it can achieve continuous dissipation of electromagnetic wave energy within a relatively wide frequency range and has the potential for broadband microwave absorption. The layered discrete dot‐cluster morphology of Ti_2_TaC_2_T_x_ reflects the gradient variation law of its dielectric properties with frequency, and there are differences in the dominant polarization and loss mechanisms in different frequency intervals. The irregular connected‐line curve of Ti_2_VC_2_T_x_ characterizes the coupling between the polarization process and the conduction process, and the synergistic effect of free electron migration and dipole rotation makes the dielectric loss change dynamically. The curve of Nb_2_CT_x_ has both the characteristics of layered dot clusters and discrete connected lines, meaning that different loss mechanisms dominate in different frequency intervals of the material, and the transition between mechanisms has a non – smooth characteristic, enabling multi – band microwave absorption. The multi – layer gradient dot – cluster curve of V_2_CT_x_ indicates that its dielectric relaxation time has a wide distribution. It can continuously convert the energy of electromagnetic waves into heat energy through multiple processes such as polarization relaxation and conduction within a wide frequency range, and has broadband microwave absorption capability. The attenuation constant (α, Figure 6f) directly correlates with absorption capacity. V_2_CT_x_ achieves the highest α (>80 Np m^−1^), consistent with its superior dielectric loss, while Nb_2_CT_x_’s weaker dielectric response results in lower α. Far‐field radiation patterns (Figure 7g–i,m–o) visualize 3D electromagnetic scattering modulation. The electromagnetic wave scattering behavior of MXenes, as revealed by radar cross‐section (RCS) analysis correlated with far‐field radiation patterns (Figure 7g–o) and RCS frequency responses (Figure 7j–r), is directly linked to their atomic composition, electronic structure, and microstructural features. The lowest RCS is exhibited by Nb_2_CT_x_ (far‐field: Figure 7n; RCS: Figure 7q), attributed to its single‐metal Nb framework, through which uniform electron distribution and optimal impedance matching with incident microwaves are enabled. Specular reflection is minimized by the symmetric 2D layered structure (Figure 7n) and consistent surface terminations, with diffusive scattering promoted and backscattering efficiently suppressed, as evidenced by its low RCS profile (Figure 7q). Lattice strain and interfacial mismatches typical of bimetallic systems are avoided by this structural uniformity, ensuring that electromagnetic waves propagate as guided modes rather than undergoing strong reflection. In contrast, superior microwave absorption is demonstrated by V_2_CT_x_ (far‐field: Figure 7o; RCS: Figure 7r), despite its slightly higher RCS than Nb_2_CT_x_, due to its unique electronic and defect‐rich architecture. Wave‐matter coupling is enhanced by its sharp V d‐orbital density of states (DOS) at the Fermi level, while abundant lattice defects induced by V's multivalence, together with surface oxygen functional groups, act as polarization centers. Dispersed scattering is shown in the far‐field pattern (Figure 7o), and reduced backscattering across angles is highlighted in the RCS response (Figure 7r), driven by defect‐mediated energy dissipation. Moderate RCS values are exhibited by Ti_2_TaC_2_T_x_ (far‐field: Figure 7i; RCS: Figure 7l) and Ti_2_VC_2_T_x_ (far‐field: Figure 7m; RCS: Figure 7p). Isotropic scattering is promoted by Ti_2_TaC_2_T_x_’s uniform lattice compression (Figure 7i) through balanced Ta/Ti orbital interactions (Figure 7l), whereas wave trapping is enhanced by Ti_2_VC_2_T_x_’s V‐induced lattice contraction and defects (Figure 7m), as evident in its suppressed RCS (Figure 7p). Higher RCS is exhibited by Ti_3_C_2_T_x_ (far‐field: Figure 7g; RCS: Figure 7j), resulting from its densely packed, ordered layers (Figure 7g), which facilitate specular reflection‐a trade‐off observable in its RCS profile (Figure 7j). The highest RCS is shown by Ti_2_NbC_2_T_x_ (far‐field: Figure 7h; RCS: Figure 7k), as lattice distortion induced by Nb's larger atomic radius (Figure 7h) weakens orbital overlap and intensifies wave reflection (Figure 7k). Overall, the RCS hierarchy, governed by atomic composition, DOS characteristics, and microstructural symmetry, is aligned with far‐field scattering patterns and RCS frequency responses, providing a visual and analytical framework for understanding how MXene properties dictate electromagnetic wave interactions. Structural uniformity is leveraged by single‐metal systems like Nb_2_CT_x_ (Figure 7n,q) for low scattering, while defect/orbital engineering is utilized by materials such as V_2_CT_x_ (Figure 7o,r) and Ti‐based MXenes to balance scattering and absorption, guiding the design of materials for radar and microwave applications.

**Figure 8:**
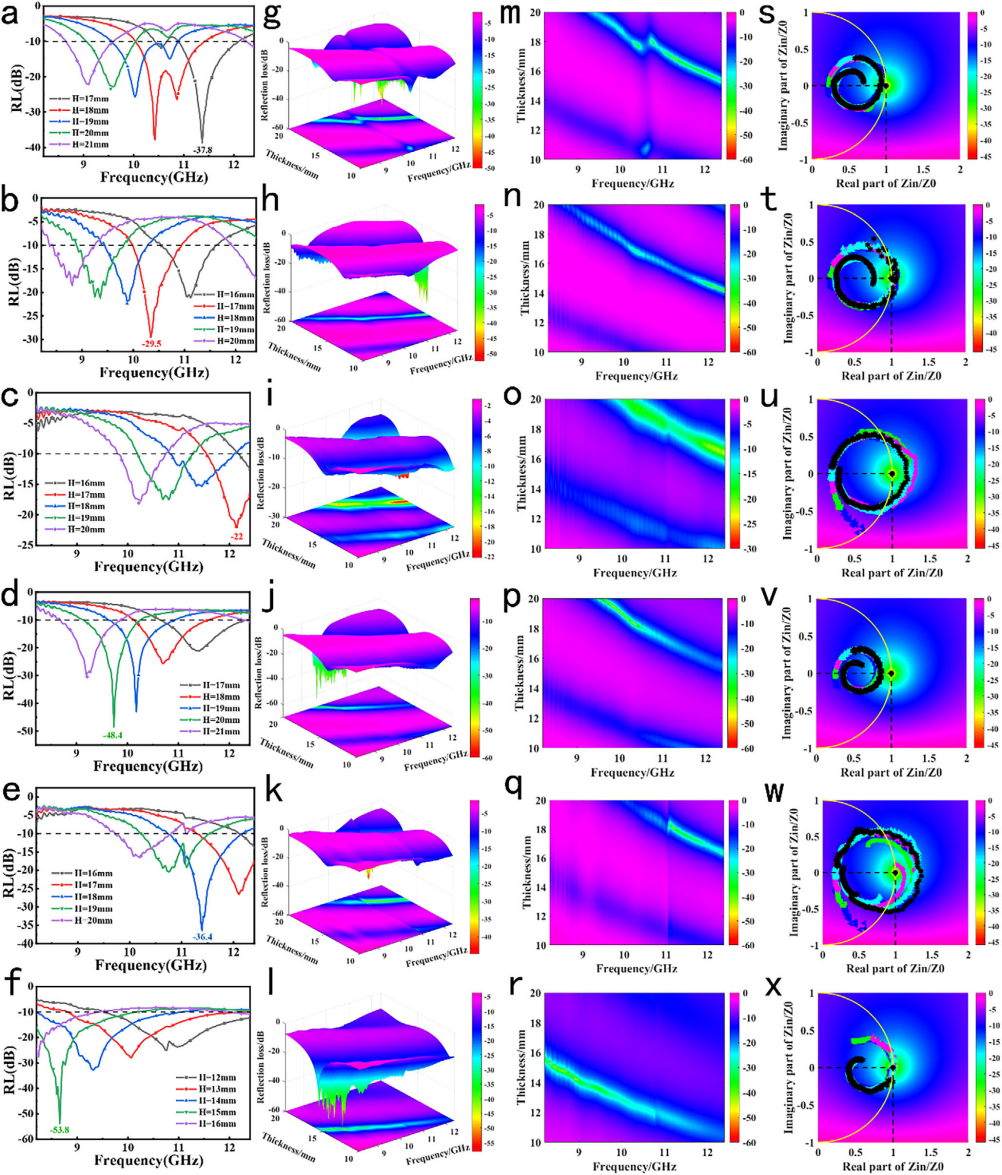
2D RL maps: a) Ti_3_C_2_T_x_; b) Ti_2_NbC_2_T_x_; c) Ti_2_TaC_2_T_x_; d) Ti_2_VC_2_T_x_; e) Nb_2_CT_x_; f) V_2_CT_x_. 3D RL maps: g) Ti_3_C_2_T_x_; h) Ti_2_NbC_2_T_x_; i) Ti_2_TaC_2_T_x_; j) Ti_2_VC_2_T_x_; k) Nb_2_CT_x_; l) V_2_CT_x_. RL contour maps: m) Ti_3_C_2_T_x_; n) Ti_2_NbC_2_T_x_; o) Ti_2_TaC_2_T_x_; p) Ti_2_VC_2_T_x_; q) Nb_2_CT_x_; r) V_2_CT_x_. Normalized impedance plots: s) Ti_3_C_2_T_x_; t) Ti_2_NbC_2_T_x_; u) Ti_2_TaC_2_T_x_; v) Ti_2_VC_2_T_x_; w) Nb_2_CT_x_; x) V_2_CT_x_.

**Figure** **8**a–f display 2D reflection loss (RL) contour maps, while Figure 8g–l present 3D RL distributions. As seen from the 2D RL plots in Figure 8f,d, V_2_CT_x_ achieved an RL as low as −53.8 dB at a thickness of 15 mm, while Ti_2_VC_2_T_x_ exhibited multiple RL values below −30 dB across the 9–12 GHz range, with the deepest loss valley at −48.4 dB. This demonstrates significantly superior wave‐absorbing performance compared to other systems. In contrast, Ti_3_C_2_T_x_ reached −38.7 dB at 17 mm thickness, and Nb_2_CT_x_ showed −36.4 dB at 18 mm thickness. V_2_CT_x_ formed prominent low RL regions across broad frequency‐thickness ranges covering 8.2–12.4 GHz. Ti_2_VC_2_T_x_ also exhibited a broad low RL coverage, outperforming Ti_3_C_2_T_x_, Nb_2_CT_x_, and other systems. The low RL (←30 dB) band of V_2_CT_x_ spanned the 8.2–11.5 GHz core frequency range, while Ti_2_VC_2_T_x_ also showed broad low RL coverage, as corroborated by the RL contour maps in Figure 8m–r. The normalized impedance circle diagrams in Figure 8s–x revealed that the impedance trajectory of V_2_CT_x_ closely approached (1,0), indicating excellent impedance matching to free space, which minimizes electromagnetic reflection and enhances incident wave absorption. The trajectory of Ti_2_VC_2_T_x_ also trended toward (1,0), showing better impedance matching than the other systems. Conversely, Ti_3_C_2_T_x_, Ti_2_NbC_2_T_x_, and others exhibited trajectories far from (1,0), leading to severe impedance mismatches and increased reflections. V_2_CT_x_ exhibits superior microwave absorption due to synergies between its V d‐orbital electronic structure, multivalence‐induced defects, and surface terminations. Sharp d‐orbital density of states at the Fermi level enhances wave‐carrier coupling, while V's multivalence generates defects and polarizable surface groups (─O/─F), activating defect and interfacial polarization losses. This dual mechanism‐carrier‐mediated and dielectric dissipation‐efficiently converts microwave energy into heat across broad frequencies, making it optimal for absorption. In comparison, Ti_3_C_2_T_x_ relied solely on conductivity loss with limited polarization contributions. Nb_2_CT_x_ suffered from insufficient carrier mobility and polarization loss due to Nb's single d‐orbital hybridization, while Ti_2_TaC_2_T_x_ experienced disrupted interlayer electron distribution due to Ta's atomic structure, further degrading performance.^[^ ^32^, ^33^, ^34^, ^35^ ^]^ Ultimately, V‐based MXenes achieved broadband, efficient absorption through optimized impedance matching, high carrier mobility, and synergistic multiple loss mechanisms, exhibiting significantly lower average RL and broader effective absorption bandwidth compared to other systems.

**Figure 9:**
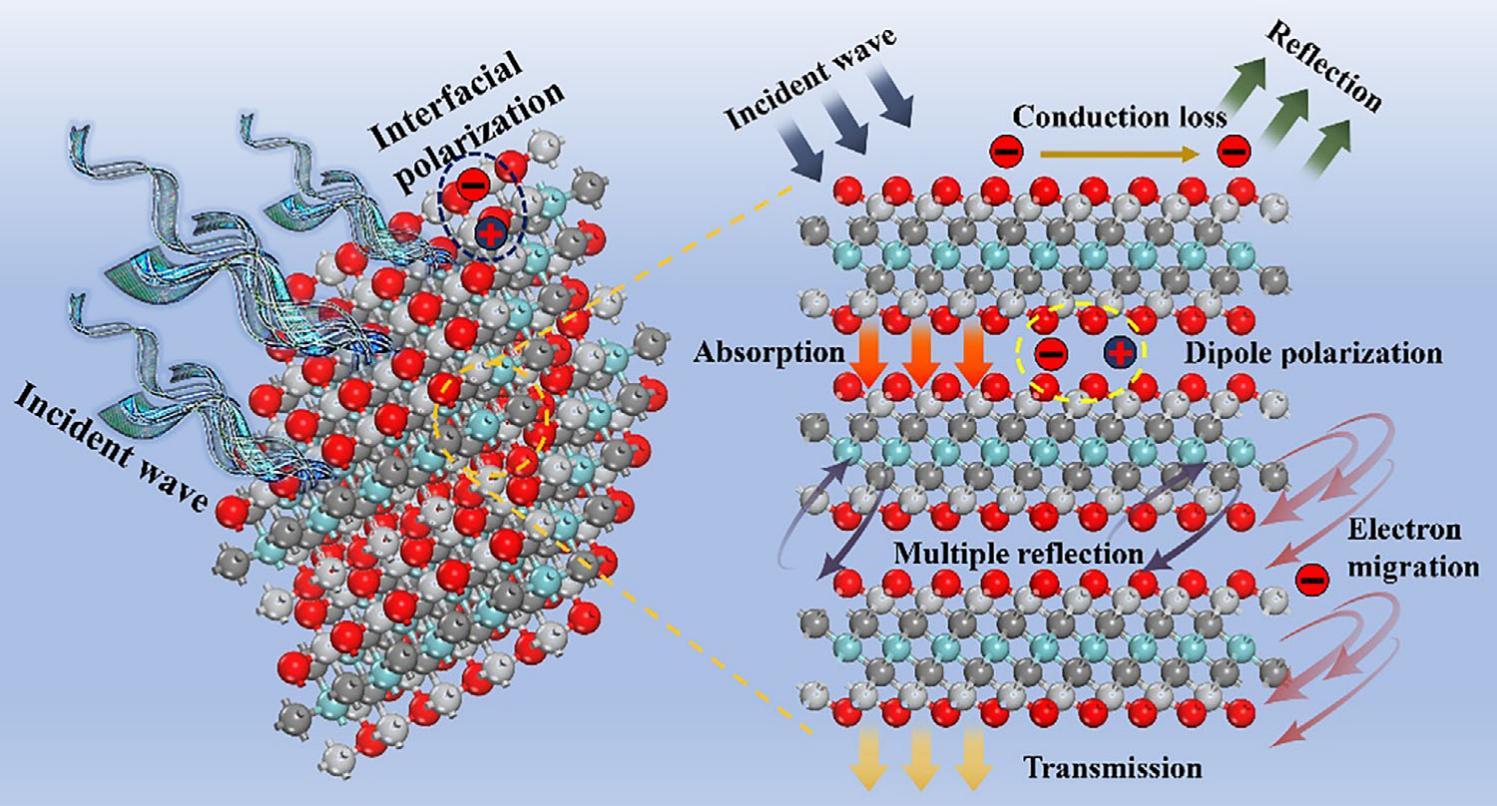
Absorption mechanism diagram of MXenes.

**Figure** **9** illustrates the absorption mechanism of MXenes. The wave‐absorbing performance of MXenes stems from the synergistic effect of multiple mechanisms. First, the conductive loss mechanism: in an alternating electromagnetic field, electrons inside MXenes migrate directionally to form a current. During this migration, collisions between electrons and lattices/impurities facilitate the conversion of electromagnetic energy into thermal energy. Second, the polarization loss mechanism, comprising interfacial polarization and dipole polarization. For interfacial polarization, charges at the interlamellar interfaces and composite‐component interfaces in MXenes are driven by the electromagnetic field to redistribute, forming electric dipoles. Their orientation and relaxation processes are accompanied by energy dissipation. Dipole polarization originates from electric dipoles formed by surface functional groups (e.g., ─OH, ─O) and internal structural defects in MXenes.^[^ ^36^, ^37^, ^38^, ^39^ ^]^ In the electromagnetic field, the orientation of these electric dipoles lags behind the electric field changes, with internal friction promoting the conversion of electromagnetic energy into thermal energy. Lastly, the multiple reflection and scattering mechanism: upon electromagnetic wave incidence on MXenes, reflections occur at internal lamellar interfaces, pore interfaces, etc., extending the propagation path and interaction time to enhance energy absorption. Meanwhile, pores and irregular structures in the material induce electromagnetic wave scattering, altering the propagation direction and further intensifying energy loss.^[^ ^40^, ^41^, ^42^, ^43^, ^44^ ^]^ The synergistic interaction of these multi‐absorption mechanisms endows MXenes with excellent electromagnetic wave absorption capability.

## 3 Conclusion

In response to the critical need for advanced microwave absorption materials to address electromagnetic interference (EMI) and enhance defense stealth technologies, this study demonstrates that atomic‐scale transition metal (TM) composition—specifically Ti, Nb, Ta, and V‐plays a pivotal role in tailoring the microwave absorption properties of MXenes. Systematic multiscale investigations reveal that the single‐metal V‐based MXene V_2_CT_x_ exhibits exceptional X‐band microwave absorption performance, achieving an ultralow RL of −53.8 dB and a broad effective absorption bandwidth of 3.4 GHz. These superior properties arise from the synergistic integration of intrinsic electronic, defect, and structural mechanisms: the multivalent V^3+^/V^4+^ d‐orbitals in V_2_CT_x_ form distinct hybridization states near the Fermi level, optimizing carrier delocalization and enhancing wave‐carrier interactions for conductive loss through efficient electron‐phonon scattering, while in Ti_2_VC_2_T_x_, V's atomic‐scale lattice contraction induces Ti─V orbital hybridization to refine charge transport and impedance matching; vanadium's inherent multivalence introduces a defect‐rich microstructure with atomic vacancies and polar surface terminations (─F/─O) in V_2_CT_x_, activating dual dielectric loss pathways via defect polarization (dipolar rotations at defective sites) and interfacial polarization (charge redistribution at interlayer interfaces) to mitigate impedance mismatch and enhance relaxational energy dissipation (As shown in FigureS1, Supporting Information, the absorption mechanism diagram of V‐based MXene); and the combination of conductive loss from V d‐orbital delocalization, polarization loss from defects and surface terminations, and optimized scattering directionality (as evidenced in far‐field patterns) enables ultrathin broadband absorption. Unlike composite‐based approaches, these properties emerge from the intrinsic electronic structure and defect characteristics of V‐based MXenes, demonstrating that TM‐mediated atomic engineering‐rather than external doping or complex composite designs‐can precisely balance conductivity, polarization, and impedance matching. This work establishes a transformative paradigm for designing MXene‐based absorbers by leveraging intrinsic orbital chemistry and defect structures, with V‐based systems offering a promising platform for ultrathin, high‐efficiency EMI shielding and stealth applications. Future studies may extend this atomic‐scale design strategy to V‐MXene heterostructures, surface termination engineering, and 3D architectures to further enhance broadband performance and scalability, underscoring the potential of atomic‐level material design in next‐generation electromagnetic management technologies.

## 4 Experimental Section

### Materials

The average particle size of MAX phase powders Ti_3_AlC_2_, Ti_2_NbAlC_2_, Ti_2_TaAlC_2_, Ti_2_VAlC_2_, Nb_2_AlC, and V_2_AlC were 38 µm. Hydrofluoric acid (HF, analytical grade, 40%) was used in this study.

### Synthesis of Ti_3_C_2_T_x_, Ti_2_NbC_2_T_x_, Ti_2_TaC_2_T_x_, Ti_2_VC_2_T_x_, Nb_2_CT_x_, and V_2_CT_x_

Ti_3_C_2_T_x_, Ti_2_NbC_2_T_x_, Ti_2_TaC_2_T_x_, Ti_2_VC_2_T_x_, Nb_2_CT_x_, and V_2_CT_x_ were synthesized via strong hydrofluoric acid etching of the corresponding Al‐containing MAX phases Ti_3_AlC_2_, Ti_2_NbAlC_2_, Ti_2_TaAlC_2_, Ti_2_VAlC_2_, Nb_2_AlC, and V_2_AlC. In a Teflon‐lined reactor, 20 mL HF was added and placed in a magnetic stirring water bath. 1 g of MAX phase powders were slowly added in small portions to the reactor while maintaining a stirring speed of 300 rpm. Reaction temperature and time varied by MAX phase type: Ti_3_AlC_2_ was etched at room temperature for 48 h; Ti_2_NbAlC_2_ at 45 °C for 48 h; Ti_2_TaAlC_2_ at 45 °C for 72 h; Ti_2_VAlC_2_ at 40 °C for 48 h; Nb_2_AlC at 50 °C for 72 h; V_2_AlC at 40 °C for 48 h. After reaction, the mixture was transferred to centrifuge tubes and centrifuged at 5000 rpm for 3 min, repeating 7–8 times until the supernatant reached neutral pH. The centrifuged solution was vacuum‐filtered, and the collected MXene powder was dried in an oven at 60 °C for 4 h.

### Material Characterization

Scanning electron microscopy (SEM) combined with energy dispersive spectroscopy (EDS) was employed to observe lamellar morphology and elemental distribution. X‐ray diffraction (XRD, Cu Kα radiation) characterized crystal structure evolution before and after etching. X‐ray photoelectron spectroscopy (XPS) analyzed surface functional groups and elemental valence states. Transmission electron microscopy (TEM) with high‐resolution TEM (HRTEM) and selected area electron diffraction (SAED) revealed crystal defects and lattice structures. Band structures and density of states (DOS) were calculated using the CASTEP module of Materials Studio software via first‐principles methods. CST electromagnetic simulation software was utilized to model radar cross‐section (RCS) characteristics.

### First‐Principles Calculation

In the first‐principles calculations, the generalized gradient approximation (GGA) with the Perdew Burk Ernzerhof (PBE) functional is employed to handle exchange – correlation interactions, carried out via Materials Studio. Prior to conducting band structure and density of states (DOS) calculations, structural optimization is performed to minimize the systems’ total energy and internal forces. The optimization convergence criteria were set as: an energy tolerance of 2 × 10^−5^ eV per atom, a max. force of 0.05 eV Å^−1^, a max. stress of 0.1 GPa, and a max. displacement of 0.002 Å. The k – point grid for optimization was 15 × 15 × 1, and a custom plane – wave cutoff of 489.8 eV was used for basis set convergence. For subsequent electronic property calculations, the self‐consistent field (SCF) tolerance was 2.0 × 10^−6^ eV per atom, wave functions were expanded with plane waves up to the set cutoff, and no +U correction was applied—since the PBE functional sufficiently described the electronic structure of the studied materials without significant on – site Coulomb interactions for target elements.

### Reflection Loss Calculation

Reflection loss (RL) of the materials was calculated using transmission line theory, with the specific formula as follows:^[^ ^45^, ^46^, ^47^ ^]^

(1) $$\mathrm{RL}\left(\mathrm{dB}\right)=20\mathrm{lg}\frac{Z_{\mathrm{in}}-Z_{0}}{Z_{\mathrm{in}}+Z_{0}}$$

(2) $$Z_{0}=\sqrt{\frac{\mu_{0}}{\varepsilon_{0}}}$$

(3) $$Z_{\mathrm{in}}=Z_{0}\sqrt{\frac{\mu_{r}}{\varepsilon_{r}}}\tanh\left[j\frac{2\pi }{c}\sqrt{\mu_{r}\varepsilon_{r}}fd\right]$$

In the formula, *f* represents the electromagnetic wave frequency, d is the material thickness, and c is the speed of light. During experiments, 15 wt.% MXene was uniformly mixed with paraffin to prepare samples measuring 22.86 × 10.16 mm in size and 2 mm in thickness. Electromagnetic parameters were measured across X‐band (8.2–12.4 GHz) using a vector network analyzer.

The attenuation constant α is calculated using the following formula:^[^ ^48^, ^49^, ^50^ ^]^

(4) $$\alpha =\frac{\sqrt{2}\pi f}{c}\sqrt{\mu^{\prime \prime }\varepsilon^{\prime \prime }-\mu^{\prime }\varepsilon^{\prime }+\sqrt{{\left(\mu^{\prime \prime }\varepsilon^{\prime \prime }-\mu^{\prime }\varepsilon^{\prime }\right)}^{2}+{\left(\mu^{\prime }\varepsilon^{\prime \prime }+\mu^{\prime \prime }\varepsilon^{\prime }\right)}^{2}}}$$

The Cole‐Cole curve serves as a visualization tool for analyzing dielectric relaxation behavior in materials, revealing internal polarization mechanisms through plots of the imaginary part (“ε”) versus real part (ε') of permittivity. Based on the Debye relaxation model, its mathematical expression is:^[^ ^51^, ^52^ ^]^

(5) $${\left(\varepsilon^{\prime \prime }\right)}^{2}+{\left(\varepsilon^{\prime }-\frac{\varepsilon_{s}+\varepsilon_{\infty }}{2}\right)}^{2}={\left(\frac{\varepsilon_{s}-\varepsilon_{\infty }}{2}\right)}^{2}$$

where *ε_s_* represents the static permittivity, and *ε_∞_* denotes the permittivity at infinite frequency.

## Conflict of Interest

The authors declare no conflict of interest.

## Supporting information



Supporting Information

## Data Availability

The data that support the findings of this study are available from the corresponding author upon reasonable request.
