# Supplementary figures and images for: Regulation of Microstructure and Absorption Properties of MXene Materials: Theoretical and Experimental
Source: Adv Sci (Weinh). 2025 Aug 11;12(41):e09994. doi: 10.1002/advs.202509994 (PMC12591198; doi:10.1002/advs.202509994)

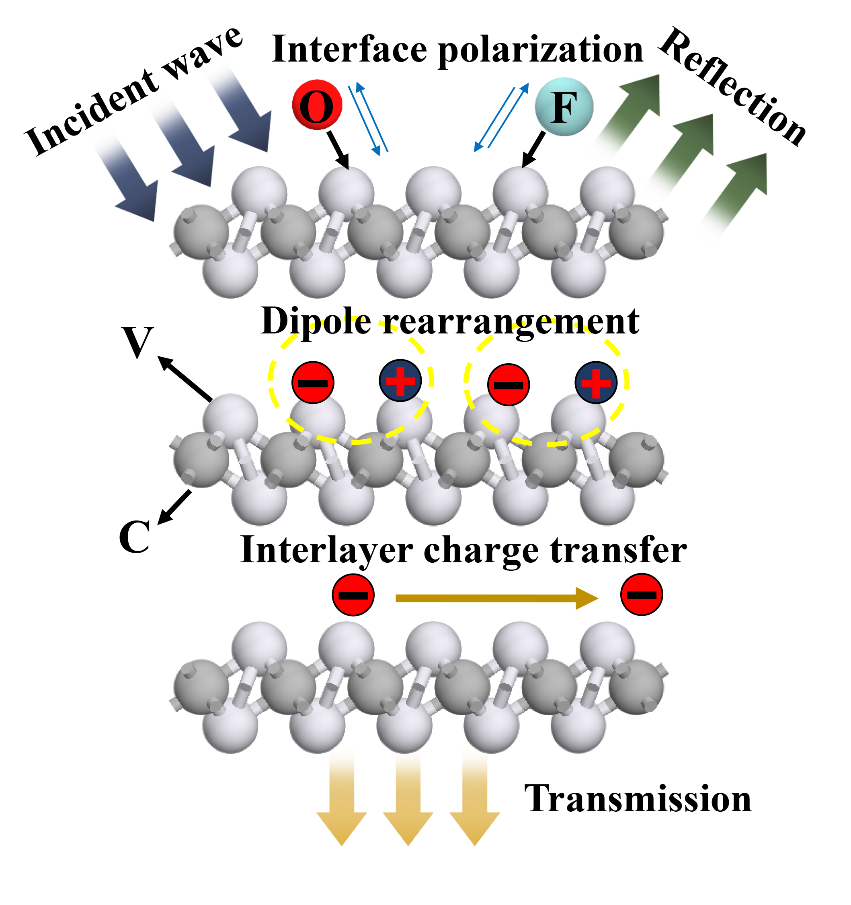


Figure S1. the absorption mechanism diagram of V-based MXene

Supplement: Supplementary file 1 — Supporting Information [file ADVS-12-e09994-s001.docx]
